# Neural and cognitive correlates of adolescent anxiety: Widespread brain network alterations, increased control costs, and visuospatial processing deficits

**DOI:** 10.1162/IMAG.a.1409

**Published:** 2026-10-30

**Authors:** Madhumitha Manjunath, Catherine Stamoulis

**Affiliations:** Department of Pediatrics, Division of Adolescence and Young Adult Medicine, Boston Children’s Hospital, Boston, MA, United States; Department of Pediatrics, Harvard Medical School, Boston, MA, United States

**Keywords:** anxiety, adolescence, brain development, functional networks, brain network controllability, cognitive function

## Abstract

This study investigated the neural substrates and cognitive correlates of adolescent anxiety, which are currently incompletely understood, in N = 3,499 youth with median (IQR) age 12.0 (1.1) years (~51.0% females), from the Adolescent Brain Cognitive Study. Resting-state fMRI, structural MRI, anxiety (scores and related disorder diagnoses), and neurocognitive data were analyzed. Mixed-effects regression models were used to examine associations between anxiety, brain characteristics (topological and morphometric properties, and regulatory (control) costs of intrinsic network dynamics), and cognitive outcomes. Females, older youth, and those with more parental mental health problems had higher anxiety (β = 0.04–1.15, 95% CI = [0.01, 2.18]). Higher resting-state connectivity and/or centrality (network importance) of the cerebellum, amygdala, and thalamus, and higher cerebellum-basal-ganglia-thalamus control costs were associated with higher anxiety (β = 0.04–0.08, 95% CI = [0.01, 0.11]). In contrast, temporoparietal and frontoparietal network efficiency and resilience (robustness) were protective for social and/or generalized anxiety disorders (adjusted odds ratio (aOR) = 0.72–0.80, 95% CI = [0.62, 0.98]). Higher cortical thickness and/or volume of the superior temporal gyrus, pars opercularis, precentral gyrus, and putamen were associated with higher odds of generalized or social anxiety disorder (aOR = 1.28–1.44, 95% CI = [1.07, 1.78]). Finally, anxiety was associated with lower response accuracy in the Little Man Task (β = -0.05, 95% CI = [-0.09, -0.01]), and attenuated positive brain-task performance associations. These findings suggest that adolescent anxiety is associated with widespread brain alterations, especially in the cerebellum-basal ganglia-thalamus network, while the organization of frontoparietal and temporoparietal networks, which play as key role in emotion regulation and social function, is protective for anxiety disorders. It is also associated with lower visuospatial processing and alterations in the networks that support it.

## 1 Introduction

Adolescence and young adulthood are sensitive periods for mental health. Common disorders often emerge in adolescence, likely as a result of complex interactions between genetic, experiential, and environmental risk factors (Kessler et al., 2005; Paus et al., 2008; Powers et al., 1989; Schlack et al., 2021; Solmi et al., 2022). In particular, anxiety, a family of disorders that encompasses generalized anxiety (GAD), social anxiety (SAD), separation anxiety, panic disorder, and various phobias, affects over 20% of adolescents in the US (Bandelow & Michaelis, 2015; CDC, 2025), and its prevalence increases with age (Cohen et al., 1993; Costello et al., 1996, 2006; Fergusson et al., 1993). The neural basis of these disorders is incompletely understood, an important gap in knowledge. To address this gap, this study investigated associations between anxiety, brain circuit organization and regional morphology, and cognitive function in a large sample of early adolescents.

The underlying drivers of youth anxiety are complex, and include genetic factors, social stressors (such as poor relationships with parents and peers, and negative family dynamics), physical environmental factors, such as noise, pollution, and unsafe neighborhoods, and negative experiences, such as trauma, abuse, and neglect (Bögels & Brechman-Toussaint, 2006; Buttazzoni et al., 2022; Calaresi et al., 2025; Cheesman et al., 2018; Chen & Qin, 2020; Colich & McLaughlin, 2022; Crandon et al., 2022; Elmore & Crouch, 2020; Huang et al., 2023; Lawrence et al., 2019; Lee et al., 2020; Lou et al., 2025; Marini et al., 2024; Marusak et al., 2023; Nanda et al., 2016; Rapee, 2012; Robinson et al., 1992; Stark et al., 1993; Teo et al., 2013; Wang et al., 2020, 2024; Waszczuk et al., 2014).

The cognitive correlates of anxiety are also complex, and include deficits in attention, memory, reward and emotional processing, decision-making, and social function (Grupe, 2017; Kilford et al., 2023; Maloney et al., 2014; Tallon et al., 2016). In adolescents, higher anxiety levels are associated with impaired emotional processing, including higher bias to threat-related inputs, poor academic performance, deficits in working memory, and risk aversion (Ducay & Alava, 2021; Dudeney et al., 2015; Manuel Prieto et al., 2023; Muris & Field, 2008; Owens et al., 2012; Rabner et al., 2024; Wolters et al., 2023). Although the mechanisms through which anxiety adversely impacts cognitive function are incompletely understood, prior studies, mostly in adults, have reported widespread circuit-level alterations underlying cognitive problems in individuals with anxiety. These include abnormally stronger intrinsic connections in the salience network, and stronger functional connections between the amygdala and the cingulo-opercular network, the striatum, default and salience networks, and the cerebellum (Jackson & Bernard, 2022; Langhammer et al., 2025; Marchand, 2010; Milad et al., 2007; Xu et al., 2021; Zharova et al., 2025). Pediatric studies have reported abnormally stronger intrinsic connections between and within limbic regions (especially the amygdala), and the salience and somatomotor networks (Lee et al., 2021; Perino et al., 2021; Sievertsen et al., 2025). They have also identified stronger connections at rest between the amygdala, the basal ganglia (especially the striatum), and the cerebellum in youth with GAD and SAD (Roy et al., 2013). Aberrant alterations in the cerebellum’s morphology and connectivity have also been reported (Chin et al., 2025), a structure that plays a key regulatory role in emotional processing and inhibitory control through its connections with the amygdala, the thalamus, and the prefrontal cortex. Similarly, abnormally stronger functional connections between the thalamus, the insula, and amygdala, but weaker connections between the thalamus and the PFC during task performance (Janiri et al., 2020), including emotional face processing (Chun et al., 2025), have also been reported.

Beyond alterations in functional connectivity, anxiety has also been associated with structural changes that have been more systematically characterized in adults compared to children. Some of these changes are common to children and adults, but others are distinct, likely reflecting inherent structural differences between the developing and developed brain. Lower gray matter volume of the anterior cingulate and insula, ventromedial and dorsolateral prefrontal cortex (PFC), superior and inferior temporal gyrus, the thalamus, the caudate, and the putamen has been reported in adults with anxiety disorders (Chen et al., 2020; Liu et al., 2024; Moon et al., 2014). Higher gray matter volume of the superior temporal gyrus and the thalamus, higher cortical thickness in the rapidly developing ventrolateral and ventromedial PFC, as well as the precentral gyrus, and lower hippocampal and amygdalar volume have been reported in adolescents (De Bellis et al., 2002; Gold et al., 2017; Hammoud et al., 2024; Liao et al., 2013; Strawn et al., 2013, 2014). Together, prior studies have identified complex, potentially bidirectional associations between anxiety and the brain. However, there are limited studies, especially in adolescents, that have integrated both brain structure and function, in order to identify associations between anxiety, functional brain circuits, and their morphological underpinnings, and relate them to cognitive processing.

Recent studies using large-scale data from the Adolescent Brain Cognitive Development (ABCD) study (Casey et al., 2018) have provided important insights into individual, environmental, and experiential correlates of adolescent anxiety. They have shown that females, youth in later pubertal stages, from low-income families, and those who spent longer times on social media and/or using screens have higher anxiety and risk of developing related disorders (Assari, 2020; Duan et al., 2025; Keyin et al., 2025; Lima Santos et al., 2026; Parker et al., 2025). Some studies have also reported brain alterations, including abnormally stronger resting-state connections between limbic regions and elements of the default, salience, frontoparietal control, and dorsal attention networks (Sievertsen et al., 2025; Thomas et al., 2024). Adolescents with lower amygdala, hippocampal, and precentral gyrus volumes have higher odds of an anxiety disorder (Hammoud et al., 2024). Those with SAD have abnormally stronger resting-state connections between the default, salience, cingulo-opercular, and retrosplenial-temporal networks (Hickson et al., 2026). Furthermore, a recent study has shown that resting-state connectivity is predictive of anxiety and depression in adolescents (Morfini et al., 2025).

Prior work has also shown that psychiatric and developmental disorders are associated with aberrant intrinsic network fluctuations (de Lacy & Calhoun, 2018; Fu et al., 2021), which are also developmental markers (Faghiri et al., 2018; Lim et al., 2025a; Sydnor et al., 2023). However, specific associations between anxiety and intrinsic network dynamics have not been previously established in adolescents. These spontaneous dynamics are regulated by endogenous (e.g., biochemical) processes that are not directly measurable in the intact human brain. In prior work, we have used a closed-loop control theoretic framework to model the feedback loop representing the reciprocal communication between these processes and the brain, and have shown that intrinsic dynamics are regulated (controlled) by a sparse set of cognitive and topological hubs, that is, highly connected regions that integrate distributed, domain-specific computations in response to cognitive demands (Lim et al., 2025b). However, associations between adolescent anxiety and controllability of intrinsic dynamics have not been investigated. Finally, most prior studies have not provided mechanistic insights into cognitive function in youth with anxiety by linking brain network and morphological abnormalities to cognitive deficits.

Leveraging large-scale neuroimaging, neurocognitive and mental health data from an ABCD adolescent sample that partly captured the heterogeneity of neural and cognitive development, this study aimed to address these important knowledge gaps. In ~3,500 youth, from the 2-year follow-up cohort (ages 11–12 years) it investigated the neural and cognitive correlates of anxiety. The study focused on early adolescence since it is a period of significant biological and social changes that increase the risk for anxiety in youth (Deardorff et al., 2007; Rapee et al., 2022). It hypothesized that anxiety is associated with morphological brain alterations underlying the atypical organization of functional circuits (especially those supporting emotional processing and social function) and intrinsic dynamics. To test this hypothesis, it investigated topological and morphometric correlates of anxiety separately, but also examined structure-function relationships. It further hypothesized that anxiety is associated with changes in the brain’s controllability and the biochemical/energy demands (‘costs’) of regulating its dynamics, likely through its impact on biochemical and metabolic processes (e.g., stress hormones) that affect intrinsic brain function. These topological, morphological, and dynamic alterations are associated with impairments in cognitive function. Finally, the study also tested the hypothesis that anxiety is not only directly associated with cognitive deficits in domains that continue to evolve in adolescence, (including memory, emotional processing, decision-making, and overall cognitive flexibility), but also moderates the brain-cognition relationship. It is associated with behavioral dysregulation but also transient biochemical changes, and longer-term hormone dysregulation, all of which can adversely modulate associations between brain and cognitive function (Blöte et al., 2014; Holder & Blaustein, 2014; Hood et al., 2015; Lo et al., 2017; Robinson et al., 2013).

## 2 Methods and Materials

The study was approved by the Boston Children Hospital Institutional Review Board.

All analyzed data are from the ABCD Release 4, which are publicly available through the NIMH Data Archive (NDA).

### 2.1 Participants

N = 3,499 youth (median (IQR) age = 12.0 (1.1) years; 50.9% females) from the ABCD 2-year follow-up assessment were included in the study, based on the following criteria: (a) availability of both anxiety and neuroimaging data (structural MRI and at least one 5-minute resting-state fMRI run) of adequate quality for analysis (less than 10% frames censored for motion based on a displacement threshold of 0.3 mm), (b) no clinical MRI findings, and (c) no diagnosis of a developmental or neuropsychiatric disorder, since these disorders adversely affect brain structural characteristics and circuit organization in disorder-specific ways (Assaf et al., 2010; Chase & Phillips, 2016; Cherkassky et al., 2006; Konrad & Eickhoff, 2010; Müller et al., 2011). Together with quality control criteria established by the ABCD, this led to the exclusion of a substantial number participants, which was necessary to ensure high-quality resting-state fMRI data for analysis of intrinsic networks and their dynamics. Demographic characteristics of participants included in and excluded from the study at the follow-up assessment were compared, and were overall statistically similar, with a slightly higher number of Hispanic participants in our sample compared to the excluded one (~22% vs 19%), and similarly for females (~51% in our vs ~46% in the excluded sample). In addition, demographic characteristics of our analytic sample were compared to those of the ABCD cohort at study entry, that is, prior to the potential introduction of attrition-related bias in the 2-year follow-up sample (see Supplementary Table S1). Our sample included slightly fewer males and slightly more females than the baseline ABCD cohort (52% males, ~48% females, compared to 49% males and ~52% females in our sample; p < 0.01), statistically similar distributions of participants across racial groups (p ≥ 0.07), a statistically similar proportion of Hispanic participants (p = 0.08), and a slightly lower proportion of non-Hispanic participants than in the ABCD cohort (~79% versus ~77% in our sample (p = 0.01). In terms of family income, our sample had a lower proportion of families with income <$50,000 and higher proportion with income >$100,000 compared to the ABCD baseline cohort (p < 0.01), but statistical similar distribution of participants in other brackets ($50,000–$100,000; p > 0.4).

### 2.2 Data

#### 2.2.1 Measures of anxiety

Anxiety raw and t-scores (standardized for age and sex) from the parent-reported Child Behaviour Checklist (CBCL) were analyzed (Wadsworth et al., 2001). Data on individual anxiety disorders, including generalized anxiety disorder (GAD), social anxiety disorder (SAD), and separation anxiety, were also extracted from the Kiddie Schedule for Affective Disorders (KSADS), and are represented by binary variables in the ABCD datasets (Hammoud et al., 2024). Finally, another binary anxiety variable was created to represent any type of anxiety, that is, 1 = any diagnosed anxiety disorder or at least subclinical scores (t ≥ 65), and 0 otherwise.

#### 2.2.2 Imaging modalities

Morphometric characteristics that had been estimated by the ABCD from fully preprocessed structural MRI, and minimally preprocessed resting-state fMRI (rs-fMRI), which had undergone initial preprocessing at the Data Analysis, Informatics & Resource Center (DAIRC) (Hagler et al., 2019) were analyzed. These data were acquired across the 21 ABCD sites, using 3.0T Siemens, GE or Phillips scanners (repetition time (TR) = 0.8 seconds; resolution = 2.4 mm isotropic), and a common imaging protocol. Minimally preprocessed rs-fMRI data (corrected for B0 distortion) that had undergone quality control checks set by the ABCD were further processed by the custom Next Generation Neural Data Analysis (NGNDA) pipeline (NGNDA, 2020). NGNDA uses tools from the Statistical Parametric Mapping (SPM) software to coregister the fMRI to the participant’s structural fMRI (T1-weighted; 1 mm isotropic, corrected for distortions associated with gradient nonlinearities; Hagler et al., 2019), and standardize to MNI space. It uses custom filtering and signal decomposition tools to correct for motion-related artifacts and censor frames based on a 0.3 mm displacement threshold, suppress cardiorespiratory and other artifacts, and harmonize the data through amplitude normalization (by a brain-specific global median), to account for signal differences between scanners and head coils (32- vs 64-channels) (Brooks et al., 2021). Voxel fMRI signals were downsampled to parcel signals (taking the median of voxels within a parcel, as an appropriate statistic given the non-normal distributions of signal amplitudes), to facilitate computationally tractable analyses. Three atlases were used for this purpose, the Schaefer cortical atlas (1,000 parcels) (Schaefer et al., 2018), the Melbourne subcortical atlas (54 parcels) (Tian et al., 2020), and the probabilistic Diedrichsen cerebellar atlas (34 parcels) (Diedrichsen et al., 2009). Topological network properties were estimated at this resolution. Similarly to our prior work (Lim et al., 2025a, 2025b), in order to conduct tractable estimations of intrinsic dynamics and control costs, the data were further downsampled at a resolution of 100 regions, based on the anatomical landmarks used to delineate the resting-state networks in Yeo et al. (2011). In addition to one high-quality rs-fMRI run (based on lowest percent of frames censored for motion (median (IQR) = 1.1 (4.0)%) and lowest median connectivity (assuming that the brain at rest is overall weakly synchronized), 2,687 participants (76.6%) had a second rs-fMRI run of adequate quality for analysis (median (IQR) = 1.6 (4.3)% of frames censored for motion). These data were analyzed as well, for replication purposes and additional exploratory analyses.

### 2.3 Topological analysis of time-compressed networks and regions

The peak signal cross-correlation was used as the estimator of pairwise connection strength. Cohort-wide statistical thresholds were estimated from connectivity matrices across all brains and fMRI runs with less than 10% of frames censored for motion (Brooks et al., 2021). The upper confidence interval of the peak cross-correlation’s moderate outlying value (median + 1.5 × IQR) was used as a global threshold to obtain adjacency matrices were estimated from connectivity matrices. Topological properties were estimated from these matrices at two spatial scales: individual regions and large-scale networks. In addition to the 17 resting-state networks delineated in Yeo et al. (2011), the reward, prefrontal, social, and central executive networks were analyzed (Blakemore, 2008; Haber & Knutson, 2010; Sherman et al., 2014). Additional circuits that combined regions implicated in anxiety in prior studies, including the amygdalo-thalamic, thalamo-basal ganglia, and amygdalo-basal ganglia circuits, were also constructed, as well as a larger network encompassing the basal ganglia (striatum, caudate, putamen, nucleus accumbens), thalamus (ventral anterior and posterior, dorsal anterior and posterior), cerebellum and amygdala (a total of 88 parcels). Network-level properties and node-level properties were estimated using the Brain Connectivity Toolbox (Rubinov & Sporns, 2010) and additional algorithms in the NGNDA pipeline. They included median connectivity within and between networks, efficiency, modularity, global clustering, topological stability, robustness and fragility, local connectedness (node degree), local clustering, and regional importance in the network (eigenvector centrality) (Brooks et al., 2021; Pasqualetti et al., 2020; Wu et al., 2011).

### 2.4 Analysis of intrinsic network dynamics and controllability

#### 2.4.1 Intrinsic brain networks and temporal fluctuations of topological properties

Time-varying resting-state connectivity matrices were estimated from fMRI signals using two approaches: (a) using a sliding window (20 frames (16.0 seconds) long, advanced by one frame) a covariance matrix was estimated, and was then transformed to a correlation matrix (the absolute correlation matrix was used for estimating time-varying topological properties) (Lim et al., 2025a); (b) each signal was transformed to its complex counterpart using the Hilbert Transform, and the instantaneous phase difference between pairs of signals was estimated. The absolute cosine of this difference was used as a measure of pairwise correlation. This estimation was done at the resolution of 1,088 parcels, and resulting connectivity matrices were then downsampled to the resolution of 100 regions. Brain-specific connectivity thresholds were then estimated as in our prior work (Lim et al., 2025a), and were used to estimate time-dependent weighted adjacency matrices. In turn, these (and corresponding binary matrices) were used to estimate time-dependent topological properties. The coefficient of dispersion (ratio of the interquartile range over the sum of the first and the third quartiles) was used as the estimator of these properties’ temporal variability).

#### 2.4.2 Controllability of intrinsic dynamics

A detailed description of the closed-loop, sparsity-promoting control framework used in the present study is described in Mitrai et al. (2023) and Lim et al. (2025b). Briefly, the brain is assumed to be a linear dynamic system that is regulated by an internal controller that exerts its control action on a sparse set of regions in order to regulate the intrinsic network dynamics of the entire brain. This framework was used to estimate feedback costs associated with each brain region and identify regions that maintain their control action over the network even at high costs. This analysis was conducted at the spatial resolution of 100 regions. In addition to region-specific feedback costs, median costs over each network of interest were also estimated.

#### 2.4.3 Temporal fluctuations of intrinsic BOLD activity

Amplitude of intrinsic signal fluctuations was estimated from each parcel rs-fMRI time series spectrum. The square root of the spectrum in the frequency range 0.01–0.1 Hz was used as a measure of fluctuation amplitude (Lim et al., 2025a; Sydnor et al., 2023). Parcel-level estimates were then downsampled to 100 regional estimates for consistency with other analyses at this resolution.

### 2.5 Morphometric properties

These properties were estimated by the ABCD from the T1-weighted MRI (Hagler et al., 2019), parcellated using the Desikan-Killiany cortical atlas (68 regions) (Desikan et al., 2006) and a probabilistic subcortical atlas (30 regions) (Fischl et al., 2002). They included cortical thickness, gray matter volume (cortical and subcortical), white matter volume (cortical), cortical surface area, and cortical and subcortical white matter intensity. The 100 regions examined in topological analyses were mapped to 98 anatomical regions in order to conduct additional secondary analyses of structure-function associations specifically in regions where anatomical and topological characteristics were correlated with anxiety.

### 2.6 Neurocognitive data

The ABCD study conducts extensive neurocognitive assessments using primarily the NIH Toolbox Cognition Battery (Luciana et al., 2018), with additional tasks that include the Little Man task, Emotional Stroop task, Delay Discounting task and Game of Dice tasks (Acker & Acker, 1982; Gelino et al., 2025; Smolker et al., 2022; Thompson et al., 2019). Although associations between anxiety, brain measures, and cognitive performance were examined across all tasks for which data were available at the 2-year follow-up, the present study focused on domains that continue to evolve in adolescence, including inhibitory control, executive function, decision-making and risk assessment, cognitive flexibility, language, and memory, assessed with the: (a) Flanker Inhibitory Control task (fully corrected for demographics t-scores), (b) Rey Auditory Verbal Learning task (RAVLT; raw scores for total correct responses), (c) Oral Reading Recognition task and (d) Peabody Picture Vocabulary task (fully corrected t-scores for both tasks), (e) Game of Dice Task (raw net score), (f) monetary Delayed Discounting task (area under the indifference points curve), (g) Little Man Task (number of correct responses and accuracy), and Emotional Stroop task (accuracy measured by the proportion of correct responses to congruent and incongruent trials, and reaction time).

### 2.7 Statistical analysis

Generalized linear and logistic mixed-effects models were used to analyze the data, and account for ABCD site effects through the inclusion of a random intercept and slope for site, and a second random intercept and slope for family. The sample included 2,794 families with one child in the study, 345 families with two children, and five families with 3 children. Linear models tested associations between anxiety scores (the dependent variable) and brain measures (and similarly for demographic characteristics), or between cognitive outcomes (the dependent variables), anxiety, and/or brain measures. Logistic models tested associations between individual anxiety disorders (or the binary anxiety variable) and brain and/or demographic parameters. In these models, the independent variable of interest (for example, brain measures) was standardized as a z-score, for better interpretation of reported adjusted odds ratios (aOR). Additional models were developed to test the moderating impact of anxiety on associations between brain measures and cognitive outcomes. These models included additive brain and anxiety parameters and an interaction term for their product.

All analyses were adjusted for baseline differences between participants using the propensity weights provided by the ABCD. In addition, all models included age, sex (male = 1, female = 2), ethnicity, race (as indicator variables), ethnicity, family income (an ordinal variable with 10 brackets), BMI z-score (Brooks et al., 2023), and pubertal stage (an ordinal variable), estimated from the parent-reported Pubertal Development scale (Petersen et al., 1988)—a 5-level scale (from pre-puberty (=1) to post-puberty (=5)), which was converted to a 4-level scale, combining those in late and post-puberty into a single category, given that very few participants (<0.1%) were in post-puberty. Number of hours per week spent on screens for purposes other than homework was also estimated from the youth screen time survey, to investigate relationships between anxiety and screen time. An additional set of models also included parental mental health, as the total problems t-score calculated from the Adult Self-Report (ASR). All models including fMRI parameters were further adjusted for time of scan (Hu et al., 2023) and percent of frames censored for motion for the analyzed run.

At each level of analysis, model results were adjusted for the False Discovery Rate (FDR) (Benjamini & Hochberg, 1995). At the network level, the FDR correction was applied across 8 topological properties of each network. At the regional level, the FDR correction was applied across properties of all regions within a particular network, assuming that p-values would likely be correlated. For each anatomical region, the correction was applied across morphometric properties. The significance level was set at α = 0.05. All data analyses were performed in the Harvard Medical School High Performance Cluster Computing, using the software MATLAB (release 2023b, Mathworks, Inc.).

## 3 Results

### 3.1 Demographic associations

Participant demographic characteristics are provided in Table 1. The race and ethnicity distribution of the sample (about 65% White and almost 80% non-Hispanic) reflected the distribution of these demographics of the larger ABCD cohort. Median (IQR) anxiety t-scores were 51 (2), and 144 (3.2%) youth had GAD, 122 (3.5%) had SAD, and 150 (4.3%) had separation anxiety. Three-hundred-ninety (11%) had at least subclinical anxiety or a diagnosis of a related disorder. Higher parental mental problems scores were associated with higher youth anxiety (β = 0.34, 95% CI = [0.31, 0.38, p < 0.01), and higher odds of GAD (aOR = 1.08, 95% CI = [1.05, 1.10], p < 0.01). Older youth, who spent more time on electronic devices, and/or with mixed race had higher anxiety (β = 0.04 to 1.15, 95% CI = [0.01, 2.18], p ≤ 0.03). Black youth and those from higher income families had lower anxiety (β = -1.44 to -0.05, 95% CI = [-2.51, -0.01], p < 0.05). Non-Hispanic youth had lower odds of GAD (aOR = 0.36, 95% CI = [0.16, 0.79], p = 0.01). Females had higher odds of having any anxiety compared to males (aOR = 1.39, 95% CI = [1.03, 1.89], p = 0.04). Additional associations and model statistics are provided in Table 2.

**Table 1.** Participant characteristics (N = 3,499).

Characteristics	Measure/categories	Statistic
		Median (IQR)
Age (Years)		12.0 (1.08)
BMI Z-Score		-0.28 (1.17)
Screen time (Hours/Week)		20.5 (18.13)
		N (%)
Sex	Male	1,715 (49.01)
	Female	1,782 (50.93)
	Missing	2 (0.06)
Race	White	2,244 (64.31)
	Black	529 (15.12)
	Asian	77 (2.20)
	Mixed Race	414 (11.73)
	Other	183 (5.23)
	Missing	52 (1.41)
Hispanic	Hispanic	768 (21.95)
	Non-Hispanic	2,696 (77.05)
	Missing	35 (1)
Family Income ($)	<25,000	344 (9.83)
	25,000 to 49,999	416 (11.89)
	50,000 to 74,999	425 (12.15)
	75,000 to 99,999	450 (12.86)
	100,000 to 199,999	1,091 (31.18)
	≥200,000	508 (14.52)
	Missing	265 (7.57)
Pubertal Stage	Prepuberty	633 (18.09)
	Early puberty	749 (21.41)
	Mid-puberty	1,130 (32.29)
	Late/Postpuberty	801 (22.89)
	Missing	186 (5.32)

**Table 2.** Statistics of mixed-effects models testing associations between demographics parameters with anxiety scores, individual anxiety disorders, or any anxiety.

Anxiety t-scores			
Parameters	β	95% CI*	p-Value
Age	0.062	[0.023, 0.101]	0.002
Race: Black	-1.440	[-2.51, -0.367]	0.009
Race: Mixed	1.151	[0.122, 2.179]	0.028
Family income	-0.050	[-0.099, -0.001]	0.045
Screen time	0.044	[0.007, 0.080]	0.021
Generalized anxiety disorder			
Parameters	aOR**	95% CI*	p-Value
Ethnicity: Hispanic	0.357	[0.161, 0.794]	0.012
Any anxiety			
Parameters	aOR**	95% CI*	p-Value
Family income	0.929	[0.865, 0.997]	0.041
Race: Mixed	2.442	[1.228, 4.859]	0.011
Sex	1.395	[1.029, 1.889]	0.031

For continuous variables, standardized regression coefficients are reported.

*CI: confidence interval; **aOR: adjusted odds ratio.

### 3.2 Associations between anxiety and brain topological properties

At the scale of individual regions, higher local connectedness (degree) and topological centrality of the cerebellum and higher amygdala degree were associated with higher anxiety (β = 0.04 to 0.07, 95% CI = [0.01, 0.11], p < 0.05). Also, higher topological clustering of the hippocampus and lower clustering of the ventral PFC and temporoparietal regions were associated with higher anxiety (β = 0.06, 95% CI = [0.02, 0.09], p = 0.01 for the hippocampus, and β = -0.07, 95% CI = [-0.12, -0.02], p < 0.05 for PFC). Furthermore, higher centrality of the parahippocampal cortex, hippocampus, and cerebellum was associated with higher odds of GAD (aOR = 1.20 to 1.32, 95% CI = [1.03, 1.54], p ≤ 0.04), whereas higher clustering in ventral PFC was associated with lower odds of GAD (aOR = 0.69, 95% CI = [0.55, 0.87], p = 0.01). Higher centrality of temporoparietal regions was associated with lower odds of SAD (aOR = 0.74, 95% CI = [0.57, 0.95], p = 0.04). Similarly, higher clustering in superior parietal regions, somatomotor regions, medial, lateral, and ventral PFC, insula, retrosplenial cortex, and bilateral cerebellum was associated with lower odds of SAD (aOR = 0.58 to 0.80, 95% CI = [0.55, 0.99], p < 0.05). The spatial distribution of these associations is shown in Figure 1.

**Fig. 1.**
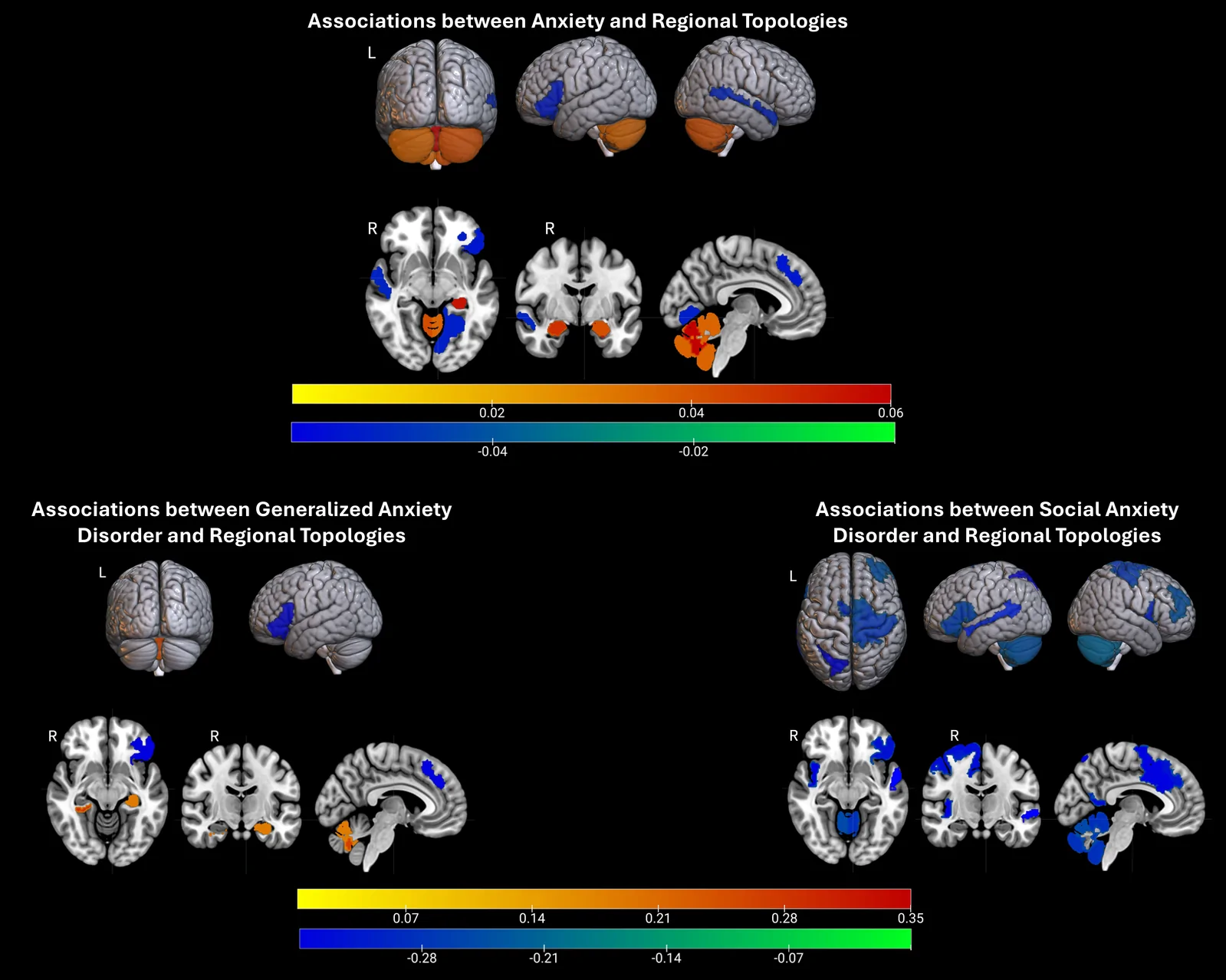
Top panel: Positive associations (yellow to red colors) between anxiety (t-scores) and regional connectedness and topological centrality (regional importance in the network), and negative associations (blue to green colors) between anxiety and regional segregation (clustering). Bottom left panel: Positive associations between Generalized Anxiety Disorder and regional centrality, and negative associations with regional segregation. Bottom right panel: Negative associations between Social Anxiety Disorder and both topological centrality and regional segregation.

At the scale of individual networks, higher connectivity between the thalamus and the rest of the brain was associated with higher anxiety (β = 0.06, 95% CI = [0.02, 0.10], p = 0.04). Higher connectivity within the thalamus was associated with higher odds of GAD (aOR = 1.34, 95% CI = [1.12, 1.59], p = 0.01; Fig. 2). Higher robustness, efficiency, clustering, and topological stability of the temporoparietal and frontoparietal networks were associated with lower odds of SAD (aOR = 0.72 to 0.79, 95% CI = [0.59, 0.97], p < 0.04; Fig. 3). Also, higher topological robustness, stability, and efficiency of the cerebellum were associated with lower odds of SAD (aOR = 0.73 to 0.80, 95% CI = [0.58, 0.97], p < 0.05). Finally, higher connectivity of the hippocampus was associated with higher odds of SAD (aOR = 1.42, 95% CI = [1.16, 1.73], p = 0.01). Model statistics are provided in Table 3. Additional associations based on analyses of the second rs-fMRI run are provided in Supplementary Table S2.

**Fig. 2.**
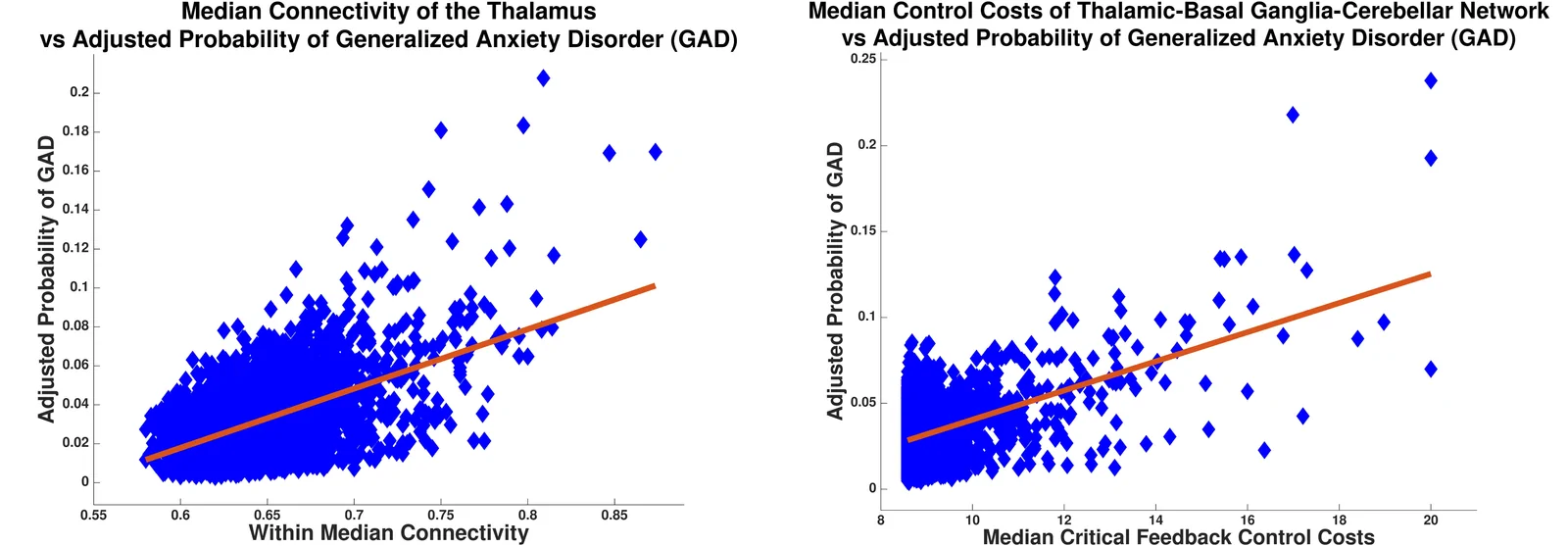
Left panel: Association between median resting-state connectivity of the thalamic network and probability of Generalized Anxiety Disorder (GAD; left panel). Right panel: Association between feedback costs associated with the controllability of the thalamic-basal ganglia-cerebellar network and probability of GAD. Probability estimates are based on logistic regression models with adjustments for demographic and imaging parameters.

**Fig. 3.**
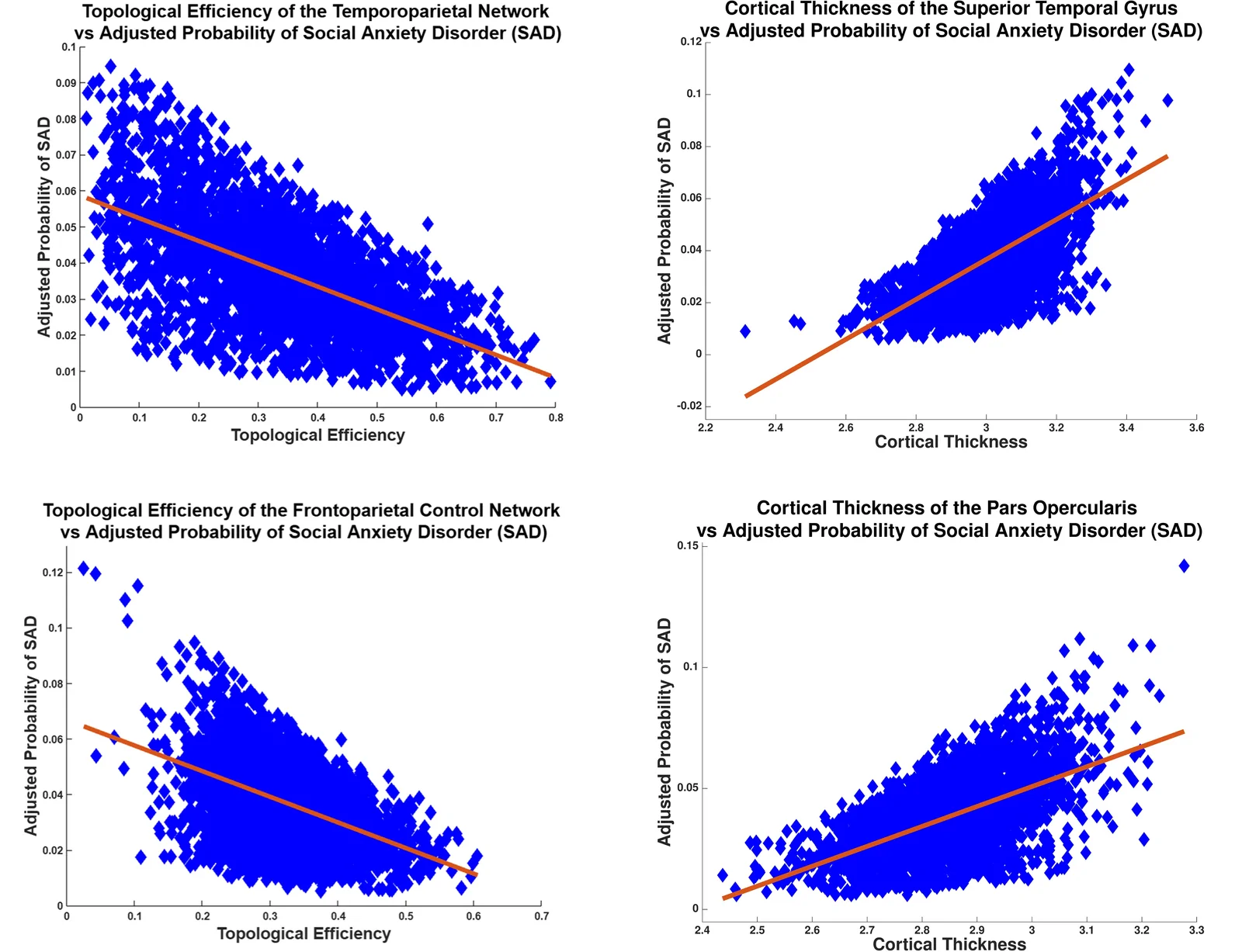
Left top and bottom panels: Negative associations between the topological efficiency of the temporoparietal (top) and frontoparietal (bottom) networks and probability of Social Anxiety Disorder (SAD). Right top panel: Positive association between thickness of the superior temporal gyrus and probability of SAD. Right bottom panel: Positive association between thickness of the pars opercularis and probability of SAD. Probability estimates are based on logistic regression models with adjustments for demographic and imaging parameters.

**Table 3.** Statistics of mixed-effects models testing associations between anxiety and network topological properties, fluctuation amplitude and median feedback control costs, based on the best-quality resting-state fMRI run.

Anxiety t-scores				
Network	Properties	β	95% CI*	p-Value
Thalamus	Median Connectivity (out)	0.061	[0.018, 0.104]	0.045
Cerebellar-Basal Ganglia (Bilateral)	Median Feedback Control Costs	0.045 to 0.046	[0.007, 0.083]	≤0.030
Cerebellar-Basal Ganglia-Thalamic (L)	Median Feedback Control Costs	0.046	[0.004, 0.089]	0.031
Cerebellar-Basal Ganglia-Amygdalo-Thalamic (Bilateral)	Median Feedback Control Costs	0.045 to 0.050	[0.002, 0.097]	≤0.039
Generalized anxiety disorder				
Network	Properties	aOR**	95% CI*	p-Value
Thalamus	Median Connectivity (in)	1.337	[1.124, 1.591]	0.009
Cerebellar-Basal Ganglia-Thalamic (L)	Median Feedback Control Costs	1.160	[1.007, 1.337]	0.039
Social anxiety disorder				
Network	Properties	aOR**	95% CI*	p-Value
Frontoparietal Control (L)	Robustness	0.771	[0.619,0.961]	0.037
	Efficiency	0.787	[0.642, 0.964]	0.037
	Topological stability	0.773	[0.621,0.965]	0.036
	Global Cluster Coefficient	0.791	[0.649, 0.963]	0.037
	Modularity	1.301	[1.067, 1.588]	0.037
Temporoparietal (L)	Robustness	0.723	[0.632,0.908]	0.0301
	Efficiency	0.772	[0.632, 0.943]	0.0301
	Global Clustering Coefficient	0.788	[0.649, 0.955]	0.032
	Topological stability	0.745	[0.597, 0.929]	0.0301
Hippocampus (R)	Median Connectivity (in)	1.416	[1.157, 1.734]	0.006
Cerebellum (bilateral)	Network Robustness	0.728 to 0.767	[0.575, 0.946]	≤0.036
	Modularity	1.324 to 1.337	[1.116, 1.586]	≤0.011
	Topological stability	0.728 to 0.739	[0.575, 0.911]	≤0.036
Cerebellum (R)	Efficiency	0.798	[0.651, 0.977]	0.046
	Global Cluster Coefficient	0.763	[0.632, 0.922]	0.014

Standardized regression coefficients are reported. All reported p-values have been adjusted for the False Discovery Rate (FDR).

*CI: confidence interval; **aOR: adjusted odds ratio.

### 3.3 Associations between anxiety and brain network controllability

At the scale of individual regions, higher anxiety was associated with higher costs of regulating (controlling) the intrinsic dynamics of the basal ganglia (β = 0.04 to 0.05, 95% CI = [0.01, 0.09], p < 0.04). Higher odds of SAD was associated with higher costs of controlling the dynamics of the parahippocampal cortex, insula, and the cerebellum and was associated with higher odds of GAD (aOR = 1.17 to 1.21, 95% CI = [1.04, 1.37], p ≤ 0.03). At the network scale, higher anxiety was associated with higher costs of controlling the cerebellum-basal ganglia, cerebellum-basal ganglia-thalamus, and cerebellum-basal ganglia-thalamus-amygdala networks (β = 0.04 to 0.05, 95% CI = [0.01, 0.10], p ≤ 0.03). In addition, higher odds of SAD were associated with higher costs of controlling cerebellum-basal ganglia-thalamus network (aOR = 1.16, 95% CI = [1.01, 1.34], p = 0.04). Model statistics are provided in Table 3. Associations between anxiety and the topological centrality of the cerebellum and costs of regulating the network that includes this structure are shown in Figure 4.

**Fig. 4.**
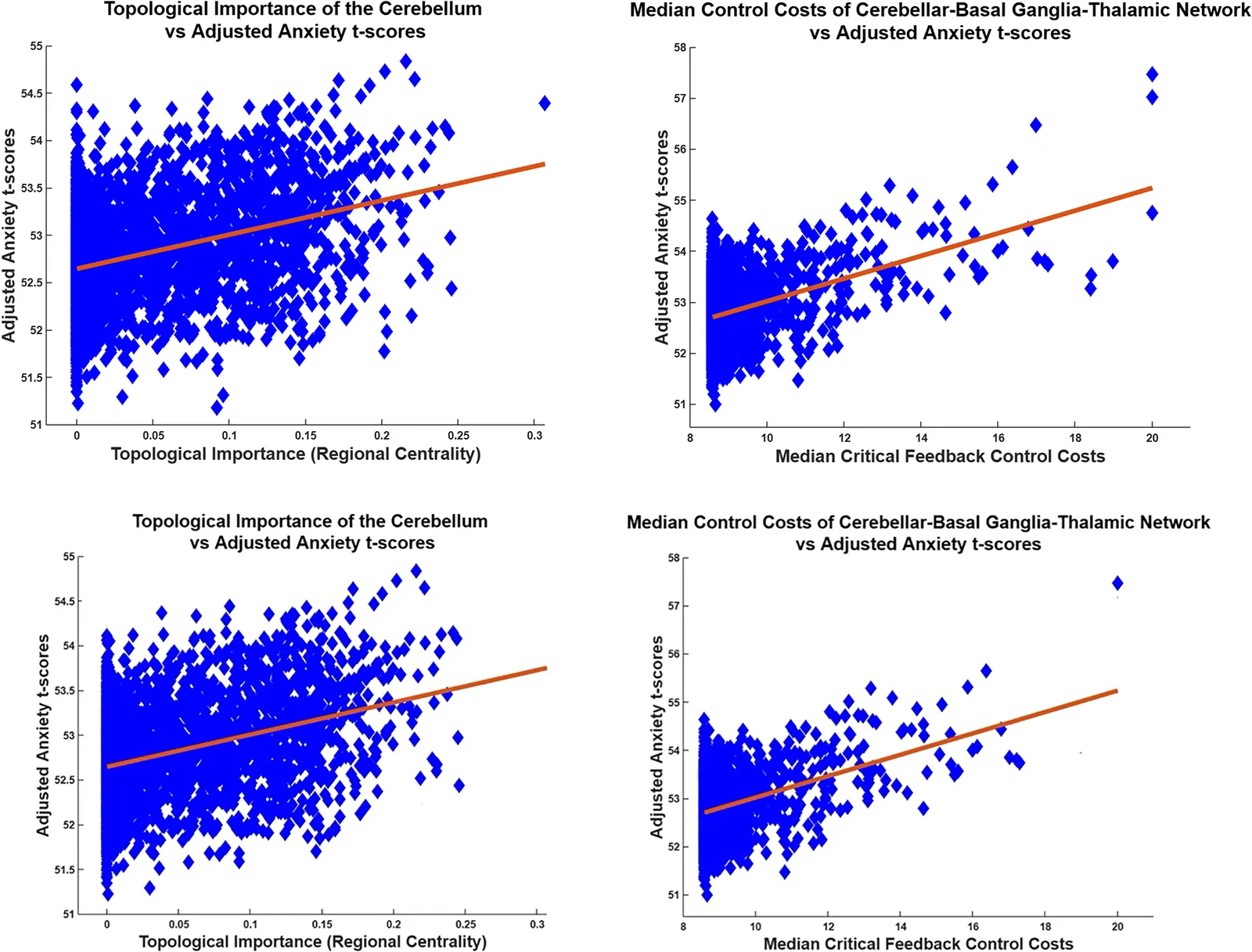
Positive associations between the topological centrality of the cerebellum and anxiety (left panel), and positive associations between cost of controlling this network and anxiety (right panel).

### 3.4 Associations between anxiety and intrinsic dynamics

Higher fluctuations in the medial and dorsal PFC, and/or frontomedial regions were associated with lower anxiety (β = -0.05 to -0.04, 95% CI = [-0.09, -0.01], p ≤ 0.04), and lower odds of GAD (aOR = 0.61 to 0.71, 95% CI = [0.49, 0.88], p ≤ 0.02). Higher fluctuations of regional connectedness (degree) and centrality of the left hippocampus, and higher degree of the right cerebellum, bilateral retrosplenial and posterior cingulate cortex, and left temporal and PFC regions were associated with lower odds of SAD (aOR = 0.73 to 0.84, 95% CI = [0.62, 0.96], p ≤ 0.03). Finally, higher temporal variability of local clustering in the right basal ganglia was associated with higher odds of GAD (aOR = 1.28, 95% CI = [1.06, 1.55], p = 0.02).

### 3.5 Associations between anxiety and structural brain properties

Higher white matter intensity in the left temporal pole and the right superior temporal gyrus was associated with higher anxiety (β = 0.05 to 0.06, 95% CI = [0.01, 0.11], p ≤ 0.03). Higher gray matter volume and surface area of the left lingual gyrus were associated with lower odds of GAD (aOR = 0.74 – 0.77, 95% CI = [0.59, 0.96], p < 0.05), whereas higher volume of the superior temporal gyrus and left putamen was associated with higher odds of SAD (aOR = 1.28 to 1.44, 95% CI = [1.03, 1.78], p ≤ 0.03). Higher cortical thickness of the left pars opercularis, left superior temporal gyrus and right precentral gyrus, and higher surface area of the entorhinal gyrus were associated with higher odds of SAD (aOR = 1.28 to 1.40, 95% CI = [1.06, 1.74], p < 0.05). Associations between likelihood of SAD and cortical thickness of superior temporal gyrus and pars opercularis, and opposite relationships between the efficiency of the networks to which these regions belong are shown in Figure 3. Finally, higher cortical thickness of the left lateral orbitofrontal cortex was associated with higher odds of any anxiety (aOR = 1.19, 95% CI = [1.05, 1.35], p = 0.02). Model statistics and additional associations are provided in Tables 4 and Supplementary Table S3.

**Table 4.** Statistics of mixed-effects models testing associations between anxiety and morphometric brain properties.

Anxiety t-scores				
Structure	Properties	β	95% CI*	p-Value
Temporal pole (L)	White matter intensity	0.052	[0.012, 0.093]	0.035
Generalized anxiety disorder				
Structure	Properties	aOR**	95% CI*	p-Value
Lingual gyrus (L)	Gray matter volume	0.738	[0.588, 0.926]	0.027
	Surface area	0.765	[0.603, 0.962]	0.044
Social anxiety disorder				
Structure	Properties	aOR**	95% CI*	p-Value
Pars opercularis (L)	Cortical thickness	1.333	[1.085, 1.636]	0.012
Superior temporal gyrus (L)	Cortical thickness	1.326	[1.069,1.645]	0.015
	Gray matter volume	1.438	[1.160, 1.782]	0.003
Putamen (L)	Gray matter volume	1.277	[1.032, 1.579]	0.025
Precentral gyrus (R)	Cortical thickness	1.365	[1.071, 1.739]	0.035
Entorhinal (R)	Surface area	1.275	[1.056, 1.540]	0.047
Any anxiety				
Structure	Properties	aOR**	95% CI*	p-Value
Lateral orbitofrontal cortex (L)	Cortical thickness	1.190	[1.048, 1.352]	0.022

Standardized regression coefficients are reported. All reported p-values have been adjusted for the False Discovery Rate (FDR).

*CI: confidence interval; **aOR: adjusted odds ratio.

In brain regions where both structural and functional properties were altered in youth with anxiety, relationships between morphometric and topological characteristics were further examined. Higher gray matter volume of the right temporal pole was associated with higher global clustering in the right limbic network (β = 0.05 to 0.06, 95% CI = [0.01, 0.10], p ≤ 0.04), and lower white matter intensity in this region was associated with lower fragility in the network (β = -0.06, 95% CI = [-0.10, -0.02], p = 0.02). Higher cortical thickness of the right superior temporal gyrus was associated with lower robustness, efficiency, and topological stability of the temporoparietal network (β = -0.05 to -0.04, 95% CI = [-0.08, -0.01], p ≤ 0.03). Higher gray matter volume and white matter intensity of the left pars opercularis was associated with higher median connectivity, robustness, and topological stability of the left ventral prefrontal network (β = 0.04 to 0.15, 95% CI = [0.01, 0.21], p ≤ 0.04). Higher white matter intensity of the left superior temporal gyrus was associated with higher connectivity, robustness, stability, and global clustering (β = 0.08 to 0.15, 95% CI = [0.03, 0.20], p ≤ 0.01) in parts of the left default mode network, and lower fragility of the parts of the left social and limbic networks overlapping with this region (β = -0.06 to -0.04, 95% CI = [-0.12, -0.01], p < 0.05) in the left social and limbic network. Higher white matter intensity of the right precentral gyrus was associated with higher median connectivity of parts of the right dorsal attention network (β = 0.09 to 0.10, 95% CI = [0.04, 0.16], p ≤ 0.03) and higher cortical thickness was associated with lower fragility in this network (β = -0.06, 95% CI = [-0.09, -0.02].

### 3.6 Associations between anxiety, brain characteristics, and cognitive outcomes

Higher anxiety was directly associated with lower scores in the Little Man Task (β = -0.05, 95% CI = [-0.09, -0.01], p = 0.02), and no other cognitive outcomes. Performance in this task was positively associated with the topological centrality of regions in the orbitofrontal, limbic, somatomotor, dorsal attention, and salience networks (β = 0.07 to 0.71, 95% CI = [0.01, 1.08], p ≤ 0.04). These associations were attenuated by higher anxiety (interaction β = -0.75 to -0.11, 95% CI = [-0.98, -0.02], p ≤ 0.04). Similarly, higher local clustering of regions in the somatomotor and salience networks was associated with better task performance (β = 0.38 to 0.57, 95% CI = [0.03, 0.92], p ≤ 0.04), but these associations were attenuated by anxiety (interaction β = -0.60 to -0.39, 95% CI = [-0.95, -0.04], p < 0.05). Higher connectivity within the bilateral somatomotor network and lower connectivity within the right social network were associated with better task performance (β = 0.48 to 0.55, 95% CI = [0.11, 0.92], p ≤ 0.02, and β = -0.57, 95% CI = [-0.95, -0.19], p = 0.02, respectively). These relationships were adversely moderated by anxiety (interaction β = -2.09 to -1.78, 95% CI = [-3.45, -0.42], p ≤ 0.02, and β = 3.01, 95% CI = [1.02, 4.99], p = 0.01, respectively). Lower segregation (modularity) of the bilateral somatomotor network, salience and social networks and left basal ganglia was associated with better performance (β = -0.49 to -0.12, 95% CI = [-0.89, -0.09], p ≤ 0.03), but these relationships were also inversely moderated by anxiety (interaction β = 0.49 to 0.57, 95% CI = [0.14, 0.95], p ≤ 0.03). Lower fragility of the somatomotor and basal ganglia networks was associated with better task performance (β = -0.39 to -0.19, 95% CI = [-0.88, -0.05], p ≤ 0.02), and anxiety inversely moderated this relationship (interaction β = 0.59 to 0.45, 95% CI = [0.08, 0.98], p ≤ 0.02). Finally, higher feedback costs associated with the insula’s control were associated with better performance β = 0.64, 95% CI = [0.23, 1.04], p = 0.01, but this relationship was attenuated by anxiety (β = -1.07, 95% CI = [-1.74, -0.40], p = 0.01).

Model statistics are provided in Table 5. Additional associations between topological properties and performance in this or other tasks were neither associated with nor moderated by anxiety.

**Table 5.** Statistics of mixed-effects moderation models testing associations between brain measures and number of correct responses in the Little Man task, and interactions between anxiety and brain measures.

Little Man Task (Sum of Correct Responses)				
Network	Parameters	β	95% CI*	p-Value
Somatomotor (Bilateral)	Median Connectivity (in)	0.481 to 0.556	[0.113, 0.917]	< 0.020
	Interaction: anxiety t-score x Median Connectivity (in)	-2.095 to -1.777	[-3.445, -0.423]	≤ 0.014
	Modularity	-0.495 to -0.493	[-0.857, -0.128]	≤ 0.015
	Interaction: anxiety t-score x Modularity	0.522 to 0.524	[0.147, 0.897]	≤ 0.012
Somatomotor (L)	Fragility	-0.390	[-0.723, -0.057]	0.024
	Interaction: anxiety t-score x Fragility	0.448	[0.078, 0.817]	0.020
Salience (Bilateral)	Modularity	-0.447 to -0.466	[-0.804, -0.092]	≤ 0.036
	Interaction: anxiety t-score x Modularity	0.468 to 0.511	[0.104, 0.857]	≤ 0.032
Social (Bilateral)	Modularity	-0.456 to -0.538	[-0.896, -0.118]	≤ 0.032
	Interaction: anxiety t-score x Modularity	0.521 to 0.571	[0.161, 0.954]	≤ 0.020
Social (R)	Median Connectivity (in)	-0.572	[-0.950, -0.194]	0.012
	Interaction: anxiety t-score x Median Connectivity (in)	3.009	[1.021, 4.997]	0.014
Basal Ganglia (L)	Modularity	-0.124	[-0.817, -0.471]	0.013
	Interaction: anxiety t-score x Modularity	0.489	[0.140, 0.838]	0.009
	Fragility	-0.194	[-0.881, -0.537]	0.010
	Interaction: anxiety t-score x Fragility	0.595	[0.209, 0.981]	0.007
Salience (Insula)l (R)	Feedback Control Costs	0.637	[0.230, 1.044]	0.007
	Interaction: anxiety t-score x Feedback Control Costs	-1.073	[-1.745, -0.401]	0.006

Standardized regression coefficients are reported. All reported p-values have been adjusted for the False Discovery Rate (FDR).

* CI: confidence interval.

## 4 Discussion

In a sample of almost 3,500 adolescents, this study investigated structural, functional and intrinsic dynamic correlates of anxiety, its relationship with cognitive function across domains, and role as a moderator of brain-cognition relationships. Higher anxiety, and generalized and social anxiety disorders were associated with structural and functional alterations of distributed brain networks and regions. These included the cerebellum, basal ganglia, thalamus, amygdala, and regions of the frontoparietal and temporoparietal networks, such as the superior temporal gyrus, the temporal pole, the insula, and the pars opercularis. Several of these regions are brain hubs that integrate multidomain information, and undergo significant topological changes in adolescence (Bagarinao et al., 2020; Oldham & Fornito, 2018; van den Heuvel & Sporns, 2013). Although our study was cross-sectional, and thus directional relationships between anxiety and brain alterations could not be established, our findings suggest that anxiety and likelihood of related disorders are associated with aberrant organization of distributed circuits and morphometric alterations of structures that play key roles in cognitive processing.

A growing number of studies have reported associations between anxiety and alterations in the cerebellum’s structural characteristics, topological organization and abnormal connections to other regions (Chin & Augustine, 2025; Lee et al., 2021; Talati et al., 2013). The cerebellum undergoes significant structural changes in adolescence. Its volume has an inverted U-shaped trajectory with a peak around age ~12 years, its subdivisions maturate at distinct rates, and it completes its development later than many other brain structures (Gaiser et al., 2024; Tiemeier et al., 2010). In addition, its widespread long-range connections with the rest of the brain also change significantly in adolescence. In particular, the cerebellum is part of multiple maturating networks that support high-level cognitive processes, including the cerebellum-cingulo-operculum network, the limbic network (a key network that connects the cerebellum to the basal ganglia through the thalamus and supports affective processing), and circuits connecting the cerebellum to the prefrontal cortex, the frontoparietal, default-mode, and temporoparietal networks (Bostan & Strick, 2018; Ceravolo et al., 2021; Clark et al., 2022; Igelström et al., 2017; Jung et al., 2022; Macher et al., 2014; O’Reilly et al., 2010; Pierce & Péron, 2020; Stockert et al., 2021). Through these extensive connections, the cerebellum plays a key role in emotional processing and regulation and social function, both of which are impacted by anxiety in adolescents (Young et al., 2019). Our findings are in agreement with prior work on relationships between the cerebellar network and anxiety, and provide insights into the neural substrates of social and emotional problems of youth with anxiety, including disorders such as SAD, and protective role of this network’s topological resilience. In addition, we show that the cost of regulating the dynamics of cerebellum-thalamus-basal ganglia network is higher in youth with anxiety. In prior work we have reported positive associations between feedback control costs and regional connectivity (Lim et al., 2025b). Thus, higher control costs in this network could be associated with abnormal connectivity and altered topological organization of the cerebellum, but also the thalamus and the basal ganglia. These structures play key roles in emotion regulation (Haber & Calzavara, 2009; Marchand, 2010). Higher regulatory costs may also reflect endogenous differences in biochemical processes, including stress hormones, that impact brain dynamics.

Higher anxiety and GAD were also associated with higher connectivity of the thalamus and stronger connections with the rest of the brain. Higher volume of the putamen was associated with higher likelihood of SAD, whereas higher efficiency and robustness of connections between the basal ganglia and the prefrontal cortex were associated with lower likelihood of SAD. These findings are in agreement with prior studies that have reported topological and structural anomalies in circuits including both structures (Xie et al., 2021).

In the frontoparietal and temporoparietal networks and their constituent structures, anxiety had both morphometric and topological correlates. Higher efficiency, topological robustness and stability of these networks were associated with lower likelihood of SAD. In contrast, higher fragility and topological segregation of the PFC, and higher cortical thickness of the pars opercularis were associated with higher likelihood of the disorder. The frontoparietal control network plays a key regulatory role in mental health, and anxiety has been associated with alterations in its organization and lower functioning of the network (Baggio et al., 2025a, 2025b; Cole et al., 2014; Kenwood et al., 2022; Sylvester et al., 2012). It undergoes significant reorganization in adolescence, to support cognitive control, and is thus vulnerable to miswiring, which can increase the risk of mental health disorders (Di Martino et al., 2014; Luna et al., 2010). Similarly, the temporoparietal network, which plays a key role in social cognition, maturates significantly in adolescence and is vulnerable to stressors (Blakemore, 2008, 2012; Mills et al., 2012; Solé-Padullés et al., 2016). Prior studies have reported associations between structural and connectivity alterations in this network and anxiety, especially SAD (Chang & Englemann, 2024; Sawalha et al., 2021; Tei et al., 2020). In our sample, higher efficiency, robustness, and topological stability of the temporoparietal network was associated with lower likelihood of SAD, whereas higher white matter intensity of the temporal pole, higher thickness, and gray matter volume of the superior temporal gyrus was associated with higher anxiety and/or likelihood of SAD. In addition, higher fragility and modularity of the social network (which overlaps with elements of the temporoparietal network and includes the superior temporal gyrus) were associated with higher likelihood of GAD and/or SAD. Together, these findings suggest that anxiety may impact widespread cortical and subcortical resting-state networks, which constitute the backbone of the brain’s neural circuitry, and play a ubiquitous role in cognitive function.

To elucidate the impact of brain alterations in youth with anxiety on cognitive function, we leveraged extensive neurocognitive assessments of the ABCD study. In agreement with prior findings, anxiety was specifically associated with worse performance (lower response accuracy) in the Little Man Task, which measures visuospatial attention and spatial reasoning, and mental rotation skills. Prior studies have reported deficits in visuospatial processing and mental rotation in individuals with anxiety, which may be more pronounced in girls (Borst et al., 2012; Kalanthroff, 2024; Lennon-Maslin & Quaiser-Pohl, 2024; Ramirez et al., 2012; Thoresen et al., 2016). Anxiety also attenuated positive associations between topological properties of the salience and the somatomotor network and response accuracy in this task. Both networks play important roles in visuospatial processing and mental rotation (Jordan et al., 2001; Toller et al., 2018; Tosoni et al., 2023; Uddin, 2016; Windischberger et al., 2003). Anxiety also inversely moderated negative associations between topological fragility and modularity of the basal ganglia and task performance, another structure involved in visuospatial processing, and negative associations between connectivity and modularity of the social network and task performance. Some of the structures and regions that form the social network are involved in visuospatial processing (Anomal et al., 2020; Blanke et al., 2005; DeLong, 2000). No other relationships between cognitive performance and anxiety were identified, which may be in part due to the relatively low prevalence of anxiety in our sample (less than 5% with any anxiety disorder, and on average anxiety scores were low).

Together, our findings suggest complex relationships between adolescent anxiety, topological and structural brain properties of interconnected regions (including brain hubs where multidomain information is integrated), and cognitive processing. These findings are in agreement with prior work but also provide a number of novel insights into the neural correlates of anxiety, especially the cerebellum-thalamus-basal ganglia circuit, which supports multidomain cognitive function, plays a key regulatory role in emotion processing, and its constituent structures maturate at distinct paces during adolescence. In addition to topological and morphometric alterations of this network, its dynamic controllability was also impacted in youth with anxiety. In prior work, we have shown that despite its extensive connections to other brain regions, the cost associated with controlling regions of this network is low, likely due to key roles of these structures in sensorimotor function that requires them to be easily regulated by endogenous processes (Lim et al., 2025b). Although youth with anxiety had aberrantly higher connectedness (and topological centrality) in these regions, and stronger connections with the rest of the brain, topological alterations may not be the only correlate of higher control costs. This network plays an important regulatory role in emotional processing, which is adversely impacted by anxiety. Our findings provide mechanistic evidence that the dysregulation of emotions in youth with anxiety may be associated with difficulty in controlling the dynamics of its underlying neural substrates. Finally, we also found that the organization of the frontoparietal and temporoparietal networks, especially higher efficiency and topological resilience, was associated with lower likelihood of SAD, which suggests that specific aspects of these networks’ wiring may be protective for mental health, in agreement with prior work, including ours (Hu & Stamoulis, 2024).

Despite its strengths, including a unique sample of almost 3,500 sample that partly captures the heterogeneity of the adolescent brain, a comprehensive investigation of neural and cognitive correlates of anxiety, including brain circuit organization, morphometric properties, intrinsic dynamics and their controllability (using a highly novel to the field approach), and an extensive set of cognitive tasks, this study also had some limitations. First, the prevalence of anxiety in the sample was relatively low, although the sample had sufficient statistical power to identify widespread neural correlates of anxiety. Second, we carefully curated our sample and excluded participants with poor quality data, and disorders that could confound our findings in ways that would be difficult to address through adjustments in statistical models. However, these decisions likely introduced a selection bias. We examined the demographic characteristics of our sample to those excluded from our analyses, as well as those of the broader ABCD cohort, and despite many similarities, there were a few relatively small differences between those samples. However, the data quality was a critical factor, especially since we conducted extensive multiscale network analyses that can be significantly affected by motion-related issues. Third, like any retrospective investigation, the study was limited by the decisions of the ABCD investigators, especially in terms of neurocognitive assessments. Nevertheless, the ABCD includes extensive cognitive tasks that measure domains that are adversely impacted by anxiety, although additional tasks on emotion processing were not available. Finally, bidirectional associations between anxiety and brain alterations could not be established. However, the ABCD study is longitudinal and additional assessments have become available. Thus, future investigations could traject the developmental trajectories of youth with anxiety over time and establish such relationships, as well as investigate the present findings as predictors of behaviors in later assessments.

## 5 Conclusions

This study has provided new insights into the neural substrates of anxiety in adolescence, a period of increased risk of psychopathologies. Its findings represent robust evidence of anxiety-related alterations in the cerebellum, basal ganglia, thalamus, frontoparietal and temporoparietal control networks and structures, which undergo significant reorganization in adolescence, and play key regulatory roles in emotional processing, social function, and decision-making. These structural and topological alterations could be examined at the level of the individual and confirmed in longitudinal studies, and could eventually become targets of precise intervention, to improve cognitive and mental health outcomes in this youth population.

## Supplementary Material

Supplementary Material

## Data Availability

The data that support the findings of this study are publicly available at the National Institute of Mental Health Data Repository: https://www.nbdc-datahub.org/. The assession number (DOI) associated with study in NDA repository is: http://dx.doi.org/10.15154/1523041. Codes used in the data analyses are available at the fMRI branch of the NGNDA: https://github.com/cstamoulis1/Next-Generation-Neural-Data-Analysis-NGNDA-/tree/NGNDA-fMRI. Statistical analysis codes specific to this manuscript are available at https://github.com/cstamoulis1/Adolescent-Anxiety
