# Supplementary material for: Neural and cognitive correlates of adolescent anxiety: Widespread brain network alterations, increased control costs, and visuospatial processing deficits
Source: Imaging Neurosci (Camb). 2026 Oct 30;4:IMAG.a.1409. doi: 10.1162/IMAG.a.1409 (PMC13726104; doi:10.1162/IMAG.a.1409)
Supplement: Supplementary file 1 [file IMAG.a.1409_supp.pdf]

SUPPLEMENTAL MATERIALS

Table S1. Age-independent participant characteristics of the ABCD cohort at study entry (N = 11,860), and statistical comparisons of these characteristics to those of the study sample.

**Characteristics**	**Measure/Categories**	**Statistic/Comparison to Study Sample**
		**Percentage (P-value)**
Sex	Male	52.15 (<0.01)[*]
	Female	47.85 (<0.01)[*]
Race	White	66.09 (0.07)
	Black	16.32 (0.09)
	Asian	2.18 (0.94)
	Mixed Race	10.9 (0.17)
	Other	4.54 (0.09)
Hispanic	Hispanic	20.6 (0.08)
	Non-Hispanic	79.03 (0.01)[*]
Family Income ($)	< 25,000	13.8 (<0.01)[*]
	25,000 to 49,999	13.4 (0.02)[*]
	50,000 to 74,999	12.6 (0.48)
	75,000 to 99,999	13.2 (0.64)
	100,000 to 199,999	27.9 (<0.01)[*]
	≥ 200,000	10.5 (<0.01)[*]

Table S2. Statistics of mixed effects models testing associations between anxiety (t-scores and individual anxiety disorders) and topological properties and median feedback control costs, estimated from the second fMRI run. Standardized regression coefficient are reported. All reported p-values have been adjusted for the False Discovery Rate (FDR). *CI: confidence interval.

**ANXIETY T-SCORES (DSM-5)**				
**Network**	**Properties**	**Beta**	**95% CI***	**P-value**
Thalamus (L)	Modularity	0.268	[0.084, 0.451]	0.037
Temporoparietal (L)		0.065	[0.028, 0.0108]	0.011
**Generalized Anxiety Disorder***				
**Network**	**Properties**	**aOR**	**95% CI***	**P-value**
Temporoparietl (L)	Modularity	1.476	[1.168, 1.865]	0.001
	Fragility	1.309	[1.051,1.631]	0.041
	Robustness	0.717	[0.539, 0.952]	0.041
	Efficiency	0.749	[0.582, 0.966]	0.041
	Topological Stability	0.722	[0.546, 0.954]	0.041
Reward (L)	Fragility	1.367	[1.142, 1.636]	0.005
Social (L)	Fragility	1.390	[1.125, 1.718]	0.018
**Social Anxiety Disorder***				
**Network**	**Properties**	**aOR**	**95% CI***	**P-value**
Salience (L)	Robustness	0.763	[0.598, 0.974]	0.046
	Modularity	1.259	[1.017, 1.559]	0.046
	Median Connectivity (out)	0.706	[0.526, 0.930]	0.036
	Efficiency	0.720	[0.571, 0.908]	0.022
	Global Clustering Coefficient	0.719	[0.575, 0.900]	0.022
	Topological Stability	0.760	[0.595, 0.972]	0.046
Reward (L)	Robustness	0.706	[0.544, 0.917]	0.021
	Fragility	1.226	[1.027, 1.464]	0.033

	Modularity	1.359	[1.085, 1.704]	0.021
	Topological Stability	0.710	[0.547, 0.923]	0.022
	Efficiency	0.721	[0.565, 0.919]	0.021
	Global Clustering Coefficient	0.761	[0.601, 0.965]	0.033
Social (L)	Modularity	1.436	[1.138, 1.812]	0.019
	Topological Stability	0.709	[0.544, 0.925]	0.026
	Robustness	0.715	[0.548, 0.932]	0.026
	Efficiency	0.720	[0.570, 0.910]	0.024
PFC (L)	Robustness	0.734	[0.569, 0.945]	≤ 0.036
	Fragility	1.265	[1.059, 1.510]	
	Modularity	1.302	[1.045, 1.622]	
	Topological Stability	0.737	[0.571, 0.952]	
	Efficiency	0.738	[0.584, 0.932]	
	Global Clustering Coefficient	0.767	[0.607, 0.969]	
Frontal-Thalamic (L)	Robustness	0.722	[0.557, 0.935]	≤ 0.031
	Modularity	1.368	[1.095, 1.709]	
	Topological Stability	0.724	[0.558, 0.940]	
	Efficiency	0.733	[0.578, 0.930]	
Frontol-Basal Ganglia-Thalamic (L)	Robustness	0.740	[0.572, 0.958]	≤ 0.047
	Modularity	1.364	[1.089, 1.709]	
	Topological Stability	0.739	[0.569, 0.959]	
	Efficiency	0.749	[0.591, 0.949]	
Fronto-Amygdalar (L)	Robustness	0.734	[0.568, 0.949]	≤ 0.041
	Efficiency	0.755	[0.596, 0.957]	
	Modularity	1.359	[1.088, 1.697]	
	Topological Stability	0.735	[0.567, 0.953]	
Fronto-Striatal-Thalamic (L)	Robustness	0.742	[0.574, 0.959]	≤ 0.047
	Efficiency	0.748	[0.590, 0.947]	
	Modularity	1.364	[1.088, 1.709]	
	Topological Stability	0.741	[0.571, 0.961]	

Table S3. Statistics of mixed effects models testing associations between anxiety (raw scores) and intrinsic dynamic and morphometrics properties. All reported p-values have been adjusted for the False Discovery Rate (FDR). *CI: confidence interval.

ANXIETY RAW SCORES				
INTRINSIC DYNAMIC PROPERTIES				
**Network**	**Properties**	**Beta**	**95% CI***	**P-value**
Default (L; medial PFC)	Fluctuation Amplitude	-0.050	[-0.089, -0.012]	0.035
Default (L; dorsal PFC)		-0.049	[-0.088, -0.012]	0.036
**MORPHOMETRIC PROPERTIES**				
**Structure**	**Properties**	**Beta**	**95% CI***	**P-value**
Superior temporal gyrus (R)	White matter Intensity	0.064	[0.017, 0.111]	0.024
Cerebellum (R)	Gray matter volume	-0.059	[-0.105, -0.013]	0.023
